# Clonal evolution from primary to locally recurrent rectal cancer reveals recurrence-acquired potentially actionable alterations in treatment-naïve tumors

**DOI:** 10.1007/s00535-026-02479-1

**Published:** 2026-08-23

**Authors:** Mamoru Uemura, Yuta Kobayashi, Hisataka Ogawa, Yurika Nakamura, Masakatsu Paku, Nobuo Takiguchi, Chikako Kusunoki, Yuki Sekido, Mitsunobu Takeda, Tsuyoshi Hata, Atsushi Hamabe, Takayuki Ogino, Norikatsu Miyoshi, Yoshinori Kagawa, Mitsuyoshi Tei, Yuichiro Doki, Hidetoshi Eguchi

**Affiliations:** 1 Department of Gastroenterological Surgery, Graduate School of Medicine, The University of Osaka, 2-2 Yamada-Oka, Suita City, Osaka 565-0871 Japan; 2 Department of Gastroenterological Surgery, Osaka International Cancer Institute, Osaka, Japan; 3 Department of Surgery, Osaka Rosai Hospital, Sakai, Japan

**Keywords:** Locally recurrent rectal cancer, Clonal evolution, Next-generation sequencing, Tumor heterogeneity, Tumors not exposed to preoperative therapy

## Abstract

**Background:**

Locally recurrent rectal cancer (LRRC) occurs in 6–10% of patients after curative surgery. It is often regarded as a simple residual disease, yet accumulating evidence suggests that LRRC is biologically distinct from primary tumors. Clonal evolution in matched primary–recurrence pairs not exposed to preoperative therapy has not been systematically investigated.

**Methods:**

We analyzed formalin-fixed paraffin-embedded tumor specimens from 15 matched pairs of primary and locally recurrent rectal cancers not exposed to preoperative therapy using targeted next-generation sequencing of 22 colorectal cancer-related genes, including TP53, KRAS, PIK3CA, MET, and FGFR family members. Tumor areas were selectively collected to ensure high tumor purity. Mutations were classified as shared or unique, and variant allele frequencies (VAFs) and clonality (VAF > 30%) were evaluated.

**Results:**

Among 415 pathogenic mutations, 12/15 pairs (80%) harbored shared driver alterations, with TP53 shared in 11 (73%). Shared mutations showed higher VAFs in recurrences than in primaries (median 57.1% vs 47.6%; *p* = 0.003). Unique mutations were modestly higher in recurrences (median 5.4% vs 4.8%; *p* = 0.048). Clonal mutations exhibited greater VAFs at recurrence (median 86.4% vs 49.8%; *p* = 0.048), while the numbers of clonal mutations were comparable (32 vs 32). Potentially actionable alterations involving MET and FGFR family genes were observed in recurrent lesions in a subset of cases, including some detected at clonal levels.

**Conclusions:**

LRRC is not merely residual disease but an evolutionarily progressed tumor entity characterized by persistence and dominance of truncal drivers with acquisition of clinically relevant alterations. Profiling recurrent lesions may guide therapy as primary tumor profiling alone may miss potentially actionable alterations present in recurrent lesions.

**Supplementary Information:**

The online version contains supplementary material available at https://doi.org/10.1007/s00535-026-02479-1.

## Introduction

Colorectal cancer (CRC) has been reported to be the third most commonly diagnosed malignancy and the second leading cause of cancer-related death worldwide in 2022 according to the GLOBOCAN statistics [1]. In rectal cancer, numerous multidisciplinary approaches, including preoperative chemoradiotherapy (CRT) and total neoadjuvant therapy (TNT), have been introduced to improve treatment outcomes. However, despite these advancements, local recurrence remains a significant clinical problem, with rates reported around 6–10% in various studies [2–5]. For the treatment of locally recurrent rectal cancer (LRRC), surgery remains the cornerstone. Nevertheless, the 5-year survival rate after surgery has been reported to be approximately 40% [6, 7]. Therefore, a comprehensive understanding of tumor biology—particularly the molecular mechanisms underlying recurrence—and the development of optimal therapeutic strategies continue to be major challenges.

LRRC has traditionally been regarded as the result of residual tumor or intraoperative dissemination of cancer cells [8, 9]. Although only limited studies have addressed this issue, our previous work [10] demonstrated that LRRCs possess biological and clinicopathological features distinct from those of primary rectal cancers. In that study, we showed that LRRC is characterized by extensive vascular invasion, an infiltrative growth pattern, and, notably, a high incidence of pulmonary metastases—findings rarely observed in primary rectal cancer. These distinct features are considered to underlie the aggressive behavior and poor prognosis of LRRC.

Clonal evolution is a fundamental principle of cancer progression [11, 12]. The development of distant metastases is thought to involve selective clonal expansion and acquisition of additional mutations, resulting in a more aggressive phenotype. However, molecular-level comparisons between treatment-naïve matched primary and recurrent tumors have rarely been performed, and no prior study has systematically characterized clonal evolution in this setting.

In this study, we performed a targeted analysis of mutational changes between treatment-naïve primary rectal tumors and their matched locally recurrent lesions using next-generation sequencing (NGS). By analyzing paired, untreated formalin-fixed paraffin-embedded (FFPE) specimens, this study aimed to clarify the evolutionary processes by which residual or disseminated cancer cells evolve into aggressive recurrent tumors, and identify molecular differences in tumor biology between primary rectal cancer and LRRC. Moreover, given that neoadjuvant therapy has become the standard of care for both advanced primary and recurrent rectal cancer, such treatment-naïve samples are increasingly difficult to obtain. This study is expected to provide valuable insights into the pathogenesis of LRRC and potential avenues for therapeutic targeting, based on intrinsically untreated tumor biology.

## Materials and methods

### Patients

Between 2000 and 2020, a total of 233 patients underwent curative-intent surgery for locally recurrent rectal cancer (LRRC) at The University of Osaka Hospital and Osaka National Hospital. Among these, only 15 cases met the following criteria and were included in this study. Eligible cases fulfilled all of the following conditions:

1. FFPE tumor specimens from the initial surgery for primary rectal cancer and FFPE tissue samples from the locally recurrent lesion were both available.
2. Neither the primary nor the recurrent tumors had received any preoperative treatment, including chemotherapy, CRT, and TNT.
3. The locally recurrent lesions were pathologically confirmed to have originated from the primary tumor, assessed by at least two board-certified pathologists with expertise in gastrointestinal pathology.
4. Informed consent was obtained from all patients for the use of clinical data and tissue samples for research purposes.

In recent years, the number of cases undergoing preoperative treatment for primary rectal cancer has been increasing in Japan [13], and since 2004, our group has adopted preoperative chemoradiotherapy (CRT) as a standard treatment protocol for LRRC [14, 15]. Therefore, cases that met the inclusion criteria for this study were limited. Clinicopathological characteristics of the matched primary and locally recurrent tumors are summarized in Tables S1 and S2.

### DNA extraction from FFPE specimens of primary rectal cancer and locally recurrent lesions

DNA was extracted from the FFPE specimens of primary rectal cancer and their corresponding locally recurrent lesions. For primary tumors, two 5-μm-thick sections were cut from the main FFPE blocks. For locally recurrent tumors, two to three 3.5-μm-thick sections were prepared and mounted onto polyethylene naphthalate (PEN) membrane slides (Leica Microsystems GmbH, Wetzlar, Germany). In LRRC specimens, the number and thickness of sections were adjusted according to the available tumor area, estimated tumor cell content, and the preparation requirements for laser microdissection and subsequent DNA extraction.

In LRRC specimens, dense fibrous stroma often obscured tumor boundaries [10]. Therefore, after deparaffinization, the slides were stained with H&E, and the tumor regions were carefully identified under microscopic observation. The histological appropriateness of the tumor areas selected for laser microdissection was confirmed in consultation with a pathologist. Two to three slides were used for laser microdissection with the LMD7000 system (Leica Microsystems), allowing selective collection of cancerous tissue. DNA was then extracted from the dissected FFPE tumor samples using the NucleoSpin® Tissue kit (TaKaRa Bio) according to the manufacturer’s protocol. In primary tumors, tumor areas were also identified based on H&E-stained slides, and representative cancer cells were selectively collected from the marked regions.

Extracted DNA was quantified using a spectrophotometer (NanoDrop ND-1000, NanoDrop Technologies, Wilmington, DE, USA), and its quality was assessed by agarose gel electrophoresis.

### DNA library preparation and targeted sequencing

DNA library preparation and deep sequencing were performed according to the manufacturer’s protocol for the Ion AmpliSeq™ Library Preparation Kit (Applied Biosystems, Life Technologies, Carlsbad, CA). A total of 10–100 ng of extracted DNA was used to construct libraries with the Ion AmpliSeq™ Kit 2.0, the Ion Xpress™ Barcode Adapters Kit, and the Ion AmpliSeq™ Colon and Lung Cancer Panel v2 (Applied Biosystems, Life Technologies). Library preparation included pre-multiplex PCR amplification, purification, adapter ligation, barcode incorporation, and multiplex PCR for clonal amplification onto beads. This cancer panel targets 504 mutational hotspot regions across the following 22 cancer-related genes:

*AKT1*, *ALK*, *BRAF*, *CTNNB1*, *DDR2*, *EGFR*, *ERBB2*, *ERBB4*, *FBXW7*, *FGFR1*, *FGFR2*, *FGFR3*, *KRAS*, *MAP2K1*, *MET*, *NOTCH1*, *NRAS*, *PIK3CA*, *PTEN*, *SMAD4*, *STK11*, and *TP53*.

The quality of the prepared libraries and enrichment was assessed using an Agilent 2100 Bioanalyzer (Agilent Technologies). The libraries were amplified onto Ion Spheres using emulsion PCR with the Ion Torrent OneTouch Template Kit and enriched on the Ion Torrent OneTouch System, following the manufacturer’s instructions. Sequencing was performed using the Ion Torrent Personal Genome Machine™ (PGM; Life Technologies) with a 318 chip.

### Mutation calling

Mutation calling was performed as previously described [16]. Base calling was conducted on the Torrent Server using Torrent Suite v5.0.2 (Thermo Fisher Scientific, Paisley, UK) under default settings and variant calling was performed using the embedded Variant Caller with high stringency settings optimized for somatic variant detection. Sequencing quality was confirmed based on coverage depth, with 93.03% of the target regions covered at ≥ 100 × and 85.29% covered at ≥ 500 ×.

Reads were subsequently processed using the coverage analysis plug-in and uploaded to the Ion Reporter v5.0 pipeline (Thermo Fisher Scientific) for variant annotation under high stringency conditions. For single-nucleotide variants (SNVs), filtering criteria included a minimum coverage of 5% for each strand, a minimum variant score of 20, a total coverage of at least 100 reads, and a strand bias threshold of 0.09. This pipeline is widely considered one of the most reliable ways for somatic mutation analysis using Ion Torrent NGS data.

After removing duplicate reads, 1904 variants were initially detected. Additional quality filters were applied to exclude low-confidence variants using the following criteria: variants in amplicons with total read depth < 100 (remaining: 1645 variants); variants with variant read depth < 40 (remaining: 1506 variants); and off-target or unannotated variants lacking gene or chromosomal band assignments (remaining: 1494 variants). Functional annotation of variants was performed using Ion Reporter and ANNOVAR [17], with variant interpretation based on multiple sources, including ClinVar [18], COSMIC [19], dbSNP (snp131, snp138), and PolyPhen-2 [20].

Variants resulting in amino acid changes—namely coding nonsynonymous SNVs and frameshift INDELs—were defined as mutations. Synonymous mutations were excluded from further analysis, resulting in 983 variants. Among these, 415 mutations were considered pathogenic if they were classified as pathogenic or likely pathogenic in ClinVar and/or COSMIC, or predicted to be deleterious by both FATHMM scores.

A full list of nonsynonymous and frameshift variants for all 30 cases—prior to pathogenic filtering—is provided in Supplementary Tables 1–30. This list was generated using ANNOVAR-based multi-annotation of high-quality variants that met stringent quality and coverage thresholds detailed above.

### Statistical analysis

All statistical tests were performed using R software (version 4.3.0). Two-group comparisons were conducted using Mann–Whitney *U* test, and categorical variables were analyzed using chi-square test. All tests were two-tailed and *p*-values < 0.05 were considered statistically significant. Figures were prepared using Adobe Illustrator CC (Adobe, San Jose, California, USA).

## Results

Using 415 pathogenic mutations identified based on the criteria described in Materials and Methods, we analyzed the mutational profiles of 22 colorectal cancer-related genes. We examined differences in mutational characteristics between primary tumors and local recurrences, focusing on the distribution, shared or unique status, variant allele frequencies, and clonality of the mutations.

### Overview of mutational landscape in primary and recurrent tumors

**Fig. 1:**
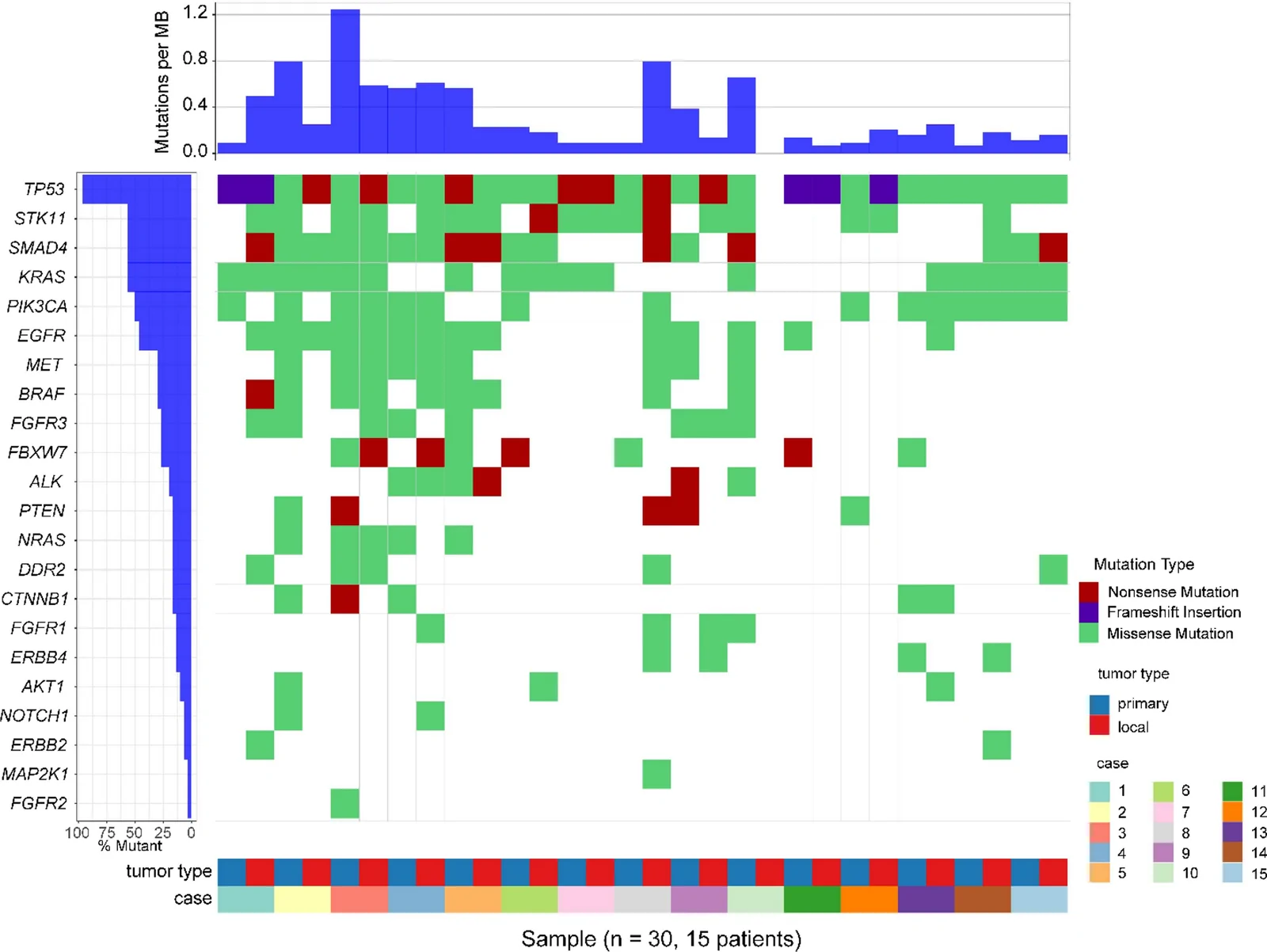
Paired waterfall plot of somatic mutations in primary tumors and local recurrences. Somatic mutations across 22 colorectal cancer-related genes are visualized in paired FFPE tumor samples from 15 patients. Each column represents either a primary tumor or a corresponding local recurrence, arranged side by side. Mutations are color-coded according to type (missense, nonsense, and frameshift insertion). Frequently mutated driver genes, such as *TP53*, *KRAS*, and *PIK3CA*, were commonly observed in both tumor types. Some recurrent tumors harbored additional alterations not present in the primary tumors, including mutations in the *FGFR* family and *MET*

The mutational profiles of primary tumors and local recurrences in each case are shown in Fig. 1. Pathogenic mutations across 22 cancer-related genes were visualized using a paired waterfall plot, displaying the mutation types (missense, nonsense, and frameshift) and their distribution across the samples. In many cases, pathogenic mutations in representative colorectal cancer-associated driver genes, such as *TP53*, *KRAS*, and *PIK3CA*, were detected either in primary tumor, recurrent tumor, or both, indicating that alterations in common colorectal cancer-associated driver genes were observed across paired primary and recurrent lesions. Although mutations in the *FGFR* family (*FGFR1*, *FGFR2*, *FGFR3*) and *MET* are relatively rare in colorectal cancer, these genes are functionally important as potential therapeutic targets. In our cohort, potentially actionable pathogenic alterations involving these genes were identified in several cases and showed discordant patterns between primary and recurrent lesions. In a subset of cases, these alterations were detected in recurrent lesions but not in the corresponding primary tumors, whereas in other cases, they were detected only in primary tumors.

### Classification of shared and unique mutations

**Fig. 2:**
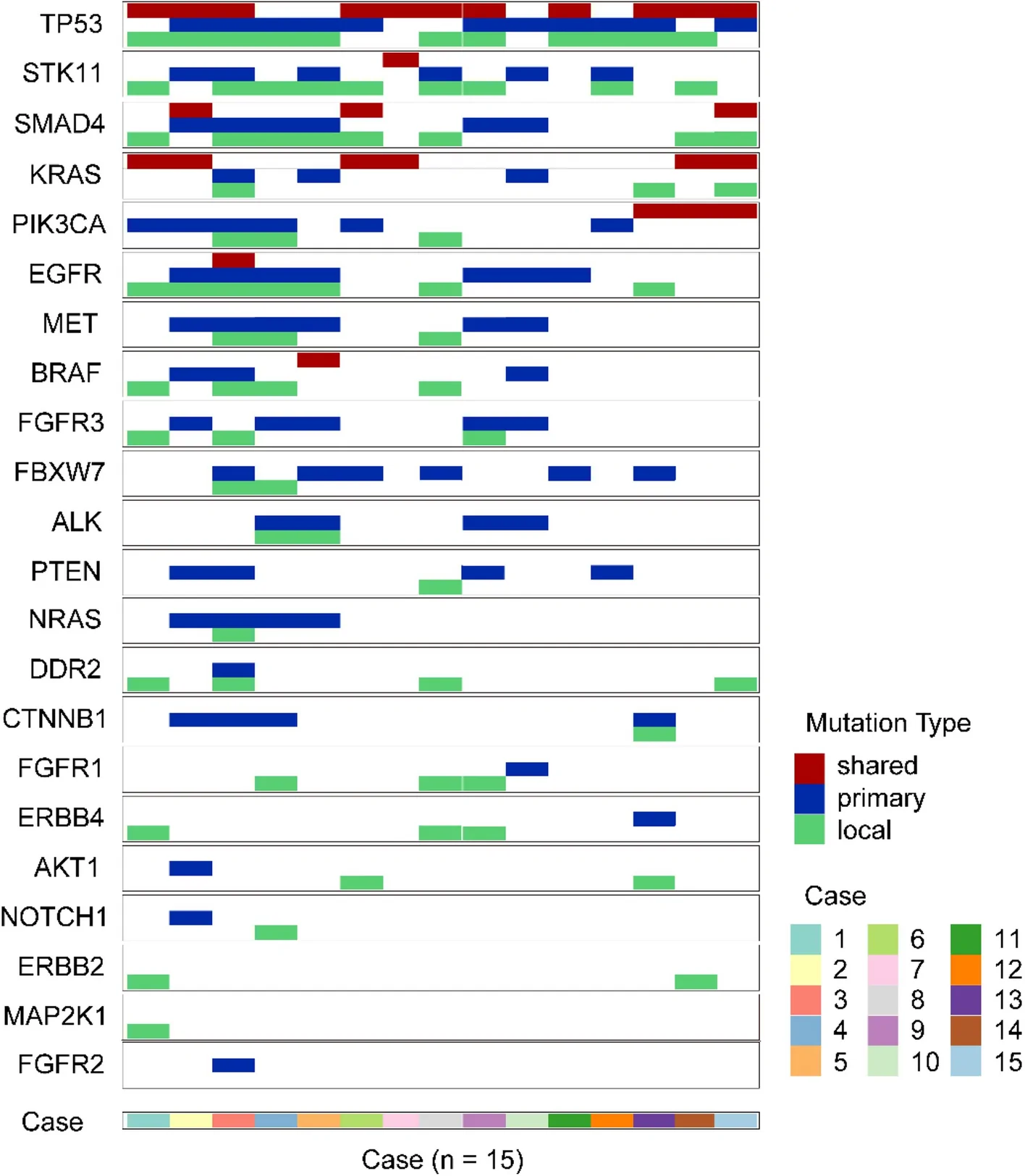
Classification of shared and unique pathogenic mutations between primary tumors and local recurrences. Pathogenic mutations detected in each of the 22 cancer-related genes were classified into three categories: shared mutations (present in both the primary and recurrent tumors), primary-unique mutations (present only in the primary tumor), and recurrence-unique mutations (present only in the local recurrence). Among the 15 paired cases, 12 (80%) harbored at least one shared mutation. Shared mutations were commonly observed in representative colorectal cancer-associated driver genes, such as *TP53*, *KRAS*, *STK11*, *SMAD4*, *PIK3CA*, *EGFR*, and *BRAF*. In contrast, many other genes harbored mutations that were detected only either in primary or recurrent tumor

Pathogenic mutations identified in both primary tumors and local recurrences were classified into two categories: shared mutations (detected in both tumors) and unique mutations (detected only in either the primary tumor or the local recurrence) shown in Fig. 2. Among the 15 cases, 12 (80%) harbored at least one shared mutation. These shared driver mutations were primarily observed in representative colorectal cancer-associated driver genes, such as *TP53*, *KRAS*, *STK11*, *SMAD4*, *PIK3CA*, *EGFR*, and *BRAF*. Notably, *TP53* was identified as a shared mutation in 11 cases (73%). Meanwhile, unique mutations, including additional *TP53* alterations, were also frequently observed either in primary or recurrent tumor.

In contrast, for many other genes, no shared mutations were observed. Instead, pathogenic alterations were found exclusively in one of the tumor sites. Thus, both shared mutations and lesion-specific unique mutations were observed across the paired primary and recurrent lesions.

### Comparison of variant allele frequencies between shared and unique mutations

**Fig. 3:**
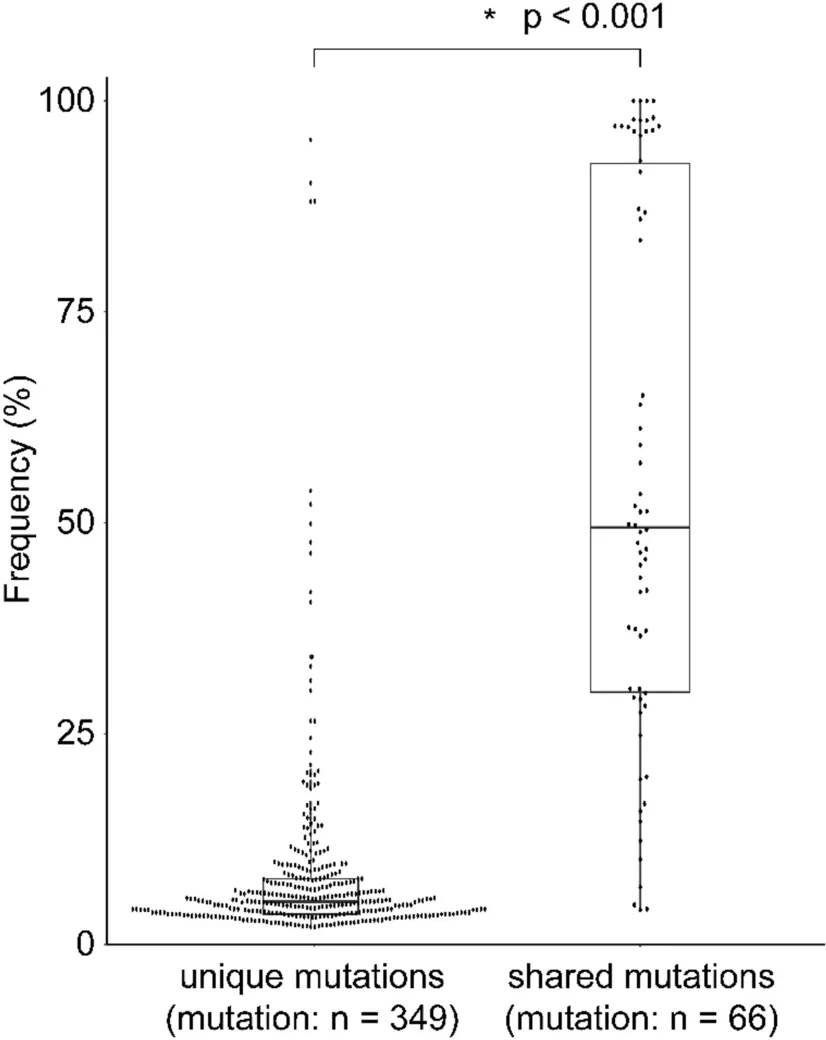
Higher variant allele frequencies in shared mutations compared to unique mutations between primary and recurrent tumors. The variant allele frequencies (VAFs) of shared and unique mutations in primary and locally recurrent tumors were compared. Shared mutations (*n* = 66) were those detected in both primary and recurrent lesions, whereas unique mutations (*n* = 349) were found in only one of the lesions. The median VAF of shared mutations was significantly higher than that of unique mutations (55.6% vs. 8.4%; *p* < 0.001)

Somatic mutations were classified into shared mutations, which were detected in both primary and locally recurrent lesions, and unique mutations, which were detected in only one of the lesions. A total of 66 mutations were identified as shared, and 349 mutations were classified as unique to either primary or recurrent tumor. The variant allele frequency (VAF) of shared mutations was significantly higher than that of unique mutations (median VAF: 49.45%, interquartile range [IQR]: 29.9–92.6% vs. 5.1%, IQR: 3.6–7.8%; p < 0.001; Fig. 3). Unique mutations included both primary- and recurrence-unique alterations.

### Increased variant allele frequencies of shared mutations in local recurrences compared with primary tumor

**Fig. 4:**
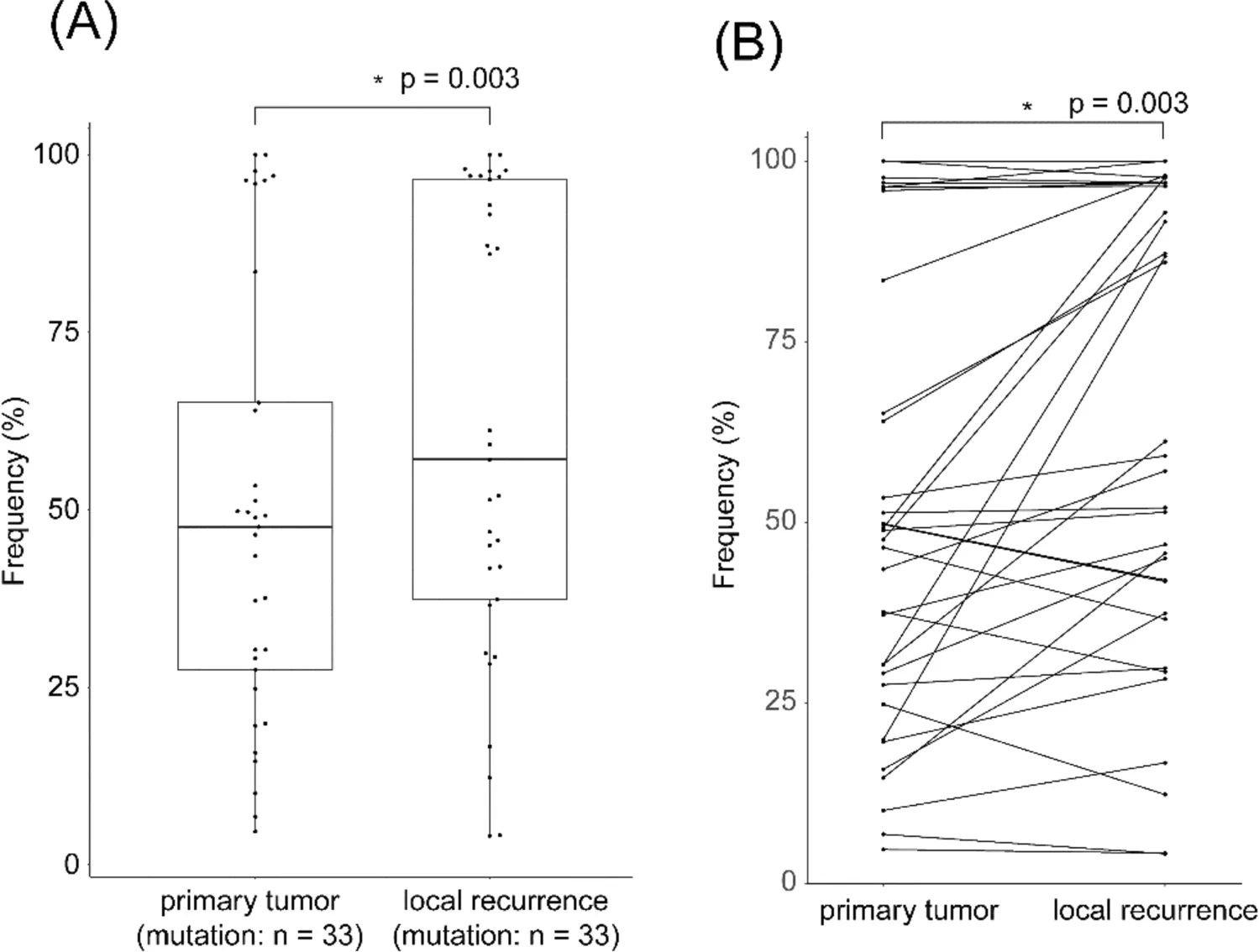
**A** Comparison of Mean Variant Allele Frequencies of Shared Mutations between Primary Tumors and Local Recurrences. The mean variant allele frequencies (VAFs) of shared pathogenic mutations were compared between primary tumors and local recurrences across the cohort. Each dot represents the VAF of a shared mutation. The median VAF was significantly higher in recurrent tumors (57.1%) than in primary tumors (47.6%) (*p* = 0.003). **B**. Paired Comparison of Variant Allele Frequencies of Shared Mutations between Primary and Recurrent Tumors. Variant allele frequencies (VAFs) of shared pathogenic mutations were compared in a paired manner between primary tumors and local recurrences from the same patients. Each line connects the VAFs of the same mutation in the primary and recurrent tumors. A statistically significant increase in VAFs was observed in the recurrent tumors (*p* = 0.003)

We compared the variant allele frequencies (VAFs) of shared pathogenic mutations between primary tumors and local recurrences. The mean VAF of shared mutations was significantly higher in local recurrences (median 57.1%, IQR: 37.4–96.5%) than in primary tumors (median 47.6%, IQR: 27.5–65.1%) (*p* = 0.003; Fig. 4A). These results suggest that clones harboring mutations associated with tumor aggressiveness may have become relatively dominant in local recurrences, potentially altering the overall tumor characteristics.

To further investigate VAF dynamics, we conducted a paired comparison of each shared mutation between the primary and recurrent tumors within the same patients. As shown in Fig. 4B, VAFs in recurrent tumors were again significantly higher than those in primary tumors (*p* = 0.003). Although some mutations exhibited decreased VAFs in the recurrent tumors, the overall trend indicated a statistically significant increase, supporting the notion that clones carrying these shared mutations became more prevalent during local recurrence.

### Unique mutations in local recurrence lesions exhibit higher variant allele frequencies than those in primary tumors

**Fig. 5:**
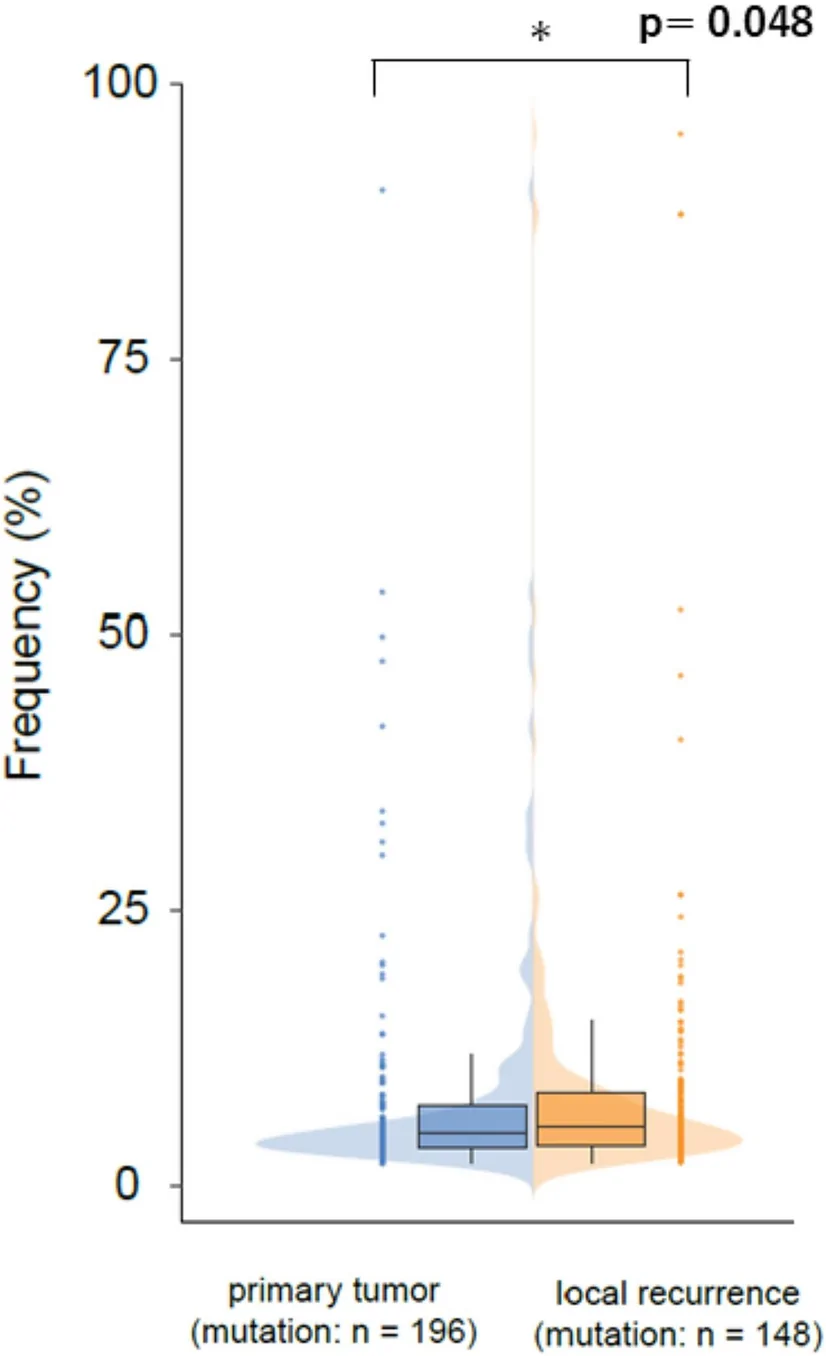
Comparison of variant allele frequencies of unique mutations between primary tumors and local recurrence lesions. The median VAFs of unique mutations were slightly higher in local recurrence lesions than in primary tumors, with a statistically significant difference (median: 5.4% vs. 4.8%, *p* = 0.048). A half violin plot was used instead of a box plot because the VAF distribution was strongly skewed toward lower values, making it difficult to visualize the group difference with conventional box plots

We compared the variant allele frequencies (VAFs) of unique mutations between primary tumors and local recurrence lesions. The median VAF of unique mutations in local recurrence lesions (median 5.4%, IQR: 3.7–8.6%) was slightly higher than that in primary tumors (median 4.8%, IQR: 3.58–7.23%), with a statistically significant difference (*p* = 0.048) (Fig. 5).

### Comparison of clonal mutation burden and composition between primary and recurrent tumors

For this analysis, somatic mutations with a variant allele frequency (VAF) greater than 30% were defined as clonal mutations. This definition is based on the theoretical framework for inferring clonal architecture using VAF [21], and is consistent with cutoffs adopted in studies of actionable driver mutations and clonal hematopoiesis in clinical oncology [22, 23].

**Fig. 6:**
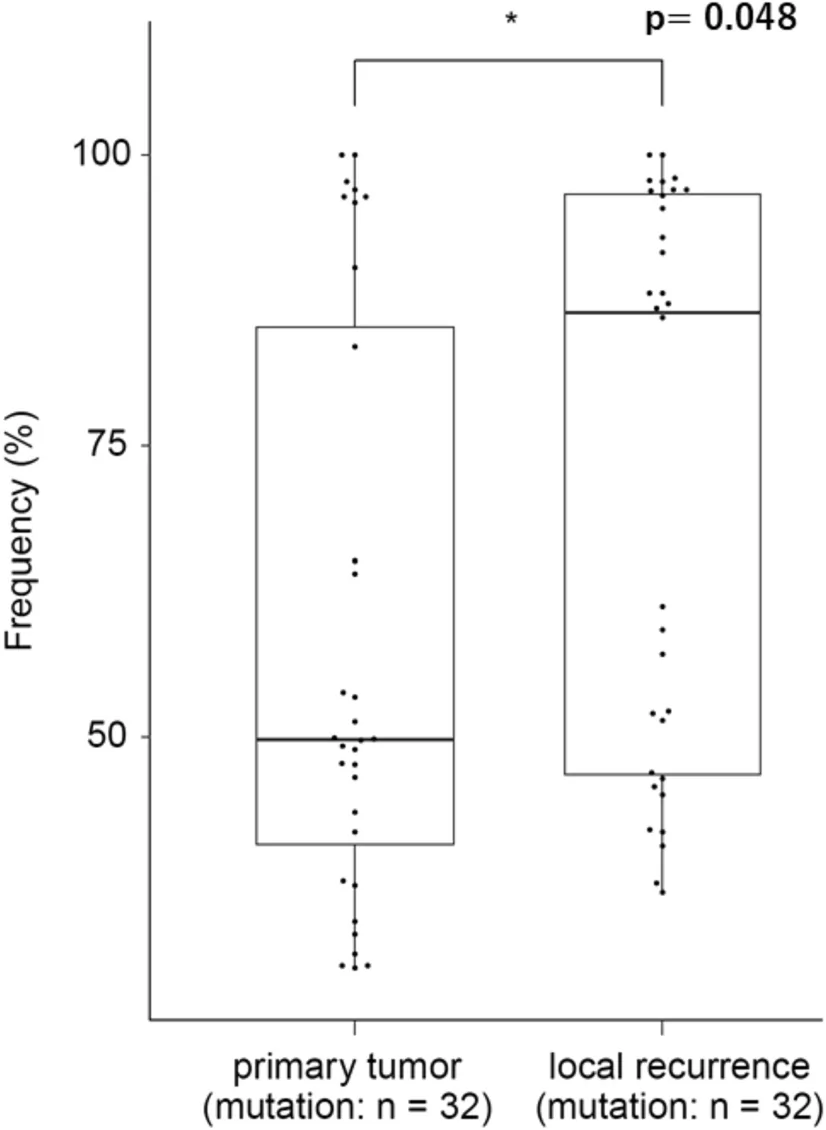
Comparison of variant allele frequency distribution of clonal mutations between primary and locally recurrent tumors. VAF distribution of clonal mutations in primary tumors and locally recurrent tumors, showing significantly higher median VAF in local recurrences than in primary tumors (median: 86.4%, IQR: 46.8–96.6% vs. 49.8%, IQR: 40.8–85.2%; *p* = 0.048), suggesting greater clonal dominance in recurrent tumors

As shown in Fig. 6, clonal mutations exhibited significantly higher VAFs in locally recurrent lesions compared to primary tumors (median 86.4%, IQR: 46.8–96.6% vs. 49.8%, IQR: 40.8–85.2%; *p* = 0.048).

**Fig. 7:**
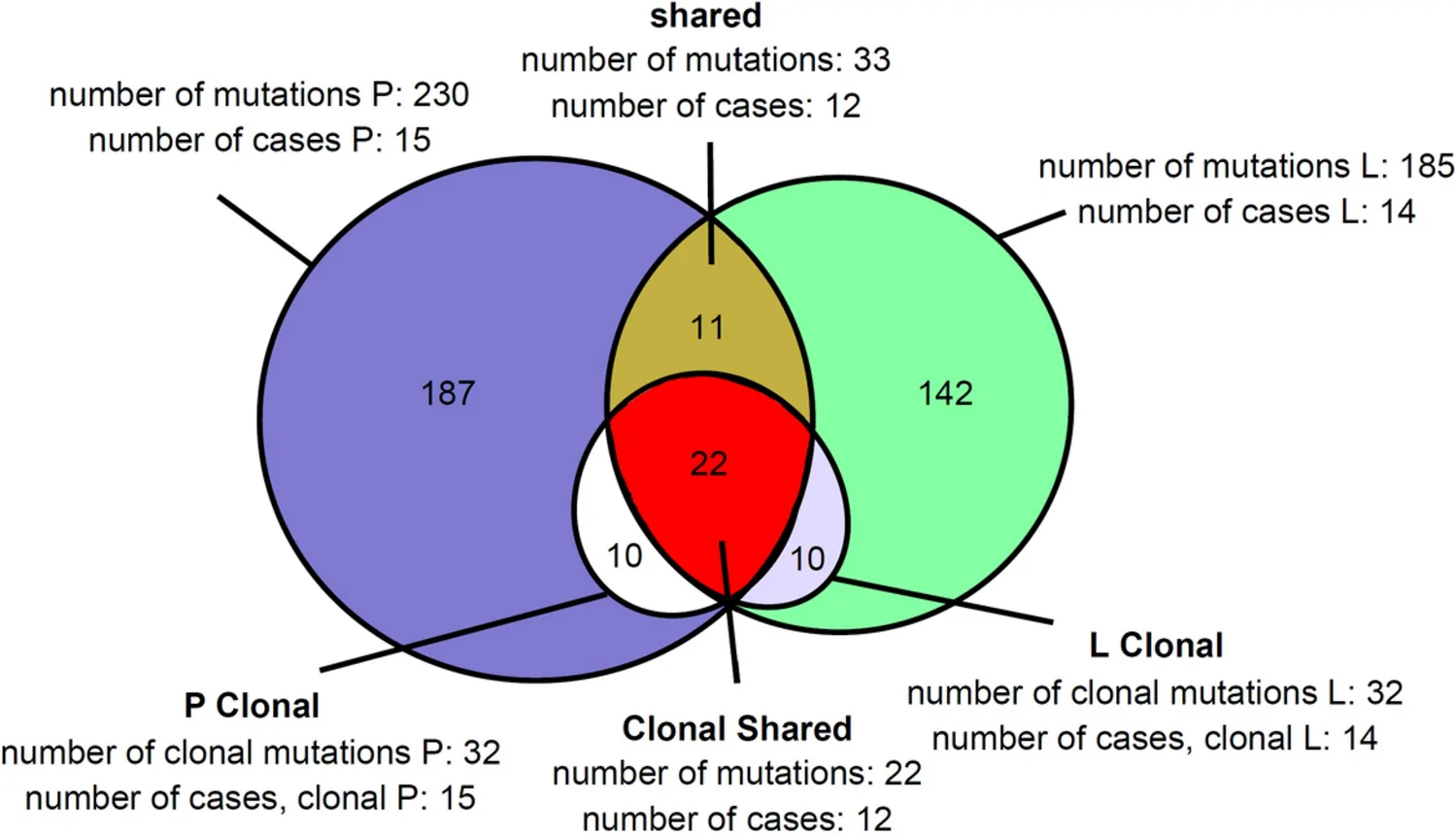
Distribution of pathogenic mutations in primary and locally recurrent tumors. A Venn diagram illustrating the distribution of pathogenic mutations in primary tumors (P) and locally recurrent tumors (L). A total of 230 pathogenic mutations were detected in the primary tumors and 185 in the recurrent tumors (total 415 pathogenic mutations across all cases). Among these, 32 clonal mutations were identified in each group. Of these clonal mutations, 22 were shared between both lesions (clonal shared), while 10 were unique to the primary tumors (P-clonal) and another 10 were unique to the recurrent tumors (L-clonal)

A Venn diagram illustrating the distribution of clonal mutations showed that 22 clonal shared mutations were detected in both primary and recurrent lesions, 10 were unique to the primary tumor (P-clonal), and 10 were unique to the recurrent lesion (L-clonal) (Fig. 7). The term “clonal shared” refers to mutations that were considered clonal in both lesions, not merely mutations that were detected in both. Although the total number of clonal mutations was the same in the primary and recurrent tumors (32 each), clonal mutations showed higher VAFs in locally recurrent lesions shown in Fig. 6.

We further examined the proportion of clonal mutations relative to the total number of mutations in both primary and recurrent lesions, but no significant difference was observed (32/185 vs. 32/230, *p* = 0.41).

## Discussion

LRRC represents a clinically significant challenge as it arises in a subset of patients even after curative-intent surgery [2–5], and curative treatment is often difficult to achieve [10, 24]. In case where local control cannot be achieved or the recurrence is deemed unresectable, its prognosis is extremely poor [25, 26]. Even when resection is feasible, LRRC often requires extensive procedures involving multi-visceral pelvic organ resection, necessitating advanced surgical techniques and multidisciplinary expertise [10, 15]. Consequently, only a limited number of specialized centers are capable of performing such complex surgeries, and detailed investigations into the tumor biology of LRRC remain scarce.

In this study, we analyzed paired FFPE tumor samples of primary and locally recurrent rectal cancers that had not undergone any neoadjuvant treatment, such as chemoradiotherapy or total neoadjuvant therapy. These treatment-naïve specimens are exceptionally valuable for assessing the natural course of tumor evolution without therapeutic influence. As neoadjuvant strategies are increasingly being adopted for both advanced primary and recurrent rectal cancer [2, 15, 27–29], the opportunity to collect such treatment-naïve paired samples is becoming extremely limited. This study therefore provides a rare and valuable opportunity to investigate clonal evolution in LRRC under unaltered biological conditions.

Cancer progression is driven by clonal evolution, whereby tumor cells acquire additional mutations and undergo selective expansion of fitter clones under environmental pressures [11]. This principle has been extensively studied in the progression from primary tumors to distant metastases [30, 31], yielding critical insights into tumor heterogeneity and therapeutic resistance. However, clonal evolution in locally recurrent rectal cancer has not been systematically investigated, and our study represents the first analysis using treatment-naïve paired FFPE specimens.

In this study, shared mutations observed in both primary and locally recurrent tumors were found to appear at higher VAFs in the recurrent lesions. These mutations are likely to be truncal mutations acquired early during tumor evolution and may function as clonal mutations essential for tumor maintenance. Unique mutations also tended to have slightly higher VAFs in the recurrent tumors, but this category should not be interpreted uniformly as late-acquired alterations because it includes both primary- and recurrence-unique mutations. Primary-unique mutations may reflect intra-tumoral heterogeneity within the primary tumor and may represent sub-clones that did not contribute to, or were not retained during, the formation of the recurrent lesion because of clonal selection or a bottleneck effect. In contrast, recurrence-unique mutations may reflect alterations that emerged during recurrence or sub-clones that expanded to detectable levels under selective pressure. Sampling effects and detection limits may also have contributed to the absence of some mutations in either lesion. Although the total number and proportion of clonal mutations were similar between primary and recurrent tumors, a marked difference was observed in their degree of infiltration. In the recurrent lesions, certain clonal mutations appeared to constitute more dominant sub-clones. Notably, several mutations were identified as clonal only in the recurrent tumors (L-clonal), supporting the emergence of new dominant sub-clones during recurrence. Collectively, these findings demonstrate that LRRC harbors a distinct clonal architecture compared with its primary counterpart. These recurrent tumors are not simply residual disease but represent evolutionarily advanced lesions with enhanced malignant potential. These observations reflect tumor progression in accordance with the theory of clonal evolution, and underscore the potential value of molecular profiling of recurrent lesions to inform therapeutic decision-making. From a clinical perspective, LRRC should not be regarded merely as a pelvic-localized disease, because it may be associated with distant metastasis and systemic failure. In our previous study of treatment-naïve LRRC resection specimens, we reported that LRRC exhibited clinicopathological features distinct from those of primary rectal cancer, including venous invasion, infiltrative growth, distant isolated cancer cells, and an association with pulmonary metastasis [10]. The present findings, including the persistence of shared driver alterations, increased VAFs of clonal mutations in recurrent lesions, and the presence of potentially actionable alterations in a subset of recurrent lesions, support the concept that LRRC represents an evolutionarily progressed tumor state with clonal evolution, and may provide a molecular background for its aggressive clinical behavior [11, 12, 30, 31]. However, because distant metastatic lesions were not analyzed in this study, we cannot conclude that these alterations directly drive distant metastatic potential.

Additionally, potentially actionable alterations involving functionally relevant but less frequently mutated genes, such as MET and members of the FGFR family, were newly detected in a subset of cases. However, these alterations should not be regarded as uniformly recurrence-associated because alterations in these genes were also detected only in primary tumors in other cases. Therefore, these genes should be interpreted as showing discordant potentially actionable alterations between primary and recurrent lesions, rather than as recurrence-associated genes. MET and FGFR family alterations are not highly frequent in metastatic colorectal cancer. However, they have been reported as potentially actionable alterations in advanced disease [32]. In particular, MET amplification, one type of MET pathway alteration, has been reported as a mechanism of acquired resistance to anti-EGFR therapy in metastatic colorectal cancer [33]. FGFR pathway alterations are also uncommon in colorectal cancer, but have been described as potentially targetable alterations across several solid tumors [34]. In addition, genomic comparison studies of primary colorectal cancers and distant metastases have shown that major driver alterations are often shared, whereas additional genomic alterations may emerge during metastatic progression [35]. Therefore, potentially actionable alterations involving MET and FGFR family genes in LRRC may partially reflect biological processes shared with clonal selection observed in distant metastases and treatment-resistant lesions. However, local recurrence and distant metastasis differ in their routes of tumor progression and microenvironmental contexts. Whereas distant metastasis requires hematogenous or lymphatic dissemination, adaptation to organ-specific metastatic niches, and in some cases, selective pressure from systemic therapy, LRRC may evolve under local selective pressures unique to the pelvis, including the postoperative pelvic environment, fibrosis, hypoxia, inflammatory changes, and local invasive growth. Therefore, because distant metastatic lesions were not analyzed in the present study, we cannot conclude that the genomic landscape of LRRC is identical to that of distant metastasis or that the evolutionary trajectory of LRRC directly mirrors that of distant metastatic disease. When present in recurrent lesions, these alterations may contribute to the malignant phenotype of recurrent lesions and suggest potential avenues for therapeutic exploration. Particularly when detected at clonal levels in recurrent lesions, these alterations could influence drug sensitivity and merit further investigation. These findings underscore the potential clinical significance of genetic profiling in recurrent tumors. Therapeutic decision-making based solely on archival primary tumor profiling may overlook potentially actionable alterations present in LRRC. In clinical practice, obtaining adequate tissue from LRRC can be challenging because recurrent lesions are often embedded in dense fibrosis and may be difficult to access safely. Accordingly, liquid biopsy approaches such as ctDNA-based profiling could be explored as a complementary strategy to capture potentially actionable alterations present in recurrent lesions and support treatment selection when tissue sampling is limited.

This study provides novel insights into the clonal evolution of LRRC by applying targeted next-generation sequencing to paired, treatment-naïve primary and recurrent FFPE tumor samples. Nevertheless, several limitations should be acknowledged. First, the sample size was relatively small (*n* = 15), reflecting the rarity of treatment-naïve pairs. Second, the retrospective design may limit generalizability. Third, one patient had undergone R1 resection at the initial surgery. In this case, the R1 status reflected microscopic margin positivity rather than macroscopic residual disease, and the positive margin site was not consistent with the main site of subsequent local recurrence. Therefore, this case was not considered to represent simple regrowth of obvious residual tumor at the positive margin. However, we cannot completely exclude the possibility that differences in recurrence mechanisms between R1 and R0 cases may have influenced the mutational profile. Fourth, although tumor areas for laser microdissection were selected on the basis of H&E-stained sections in consultation with a pathologist, intra-tumoral heterogeneity and sampling effects cannot be completely excluded, particularly in LRRC specimens with dense fibrosis. Fifth, our targeted 22-gene panel cannot capture the entire mutational landscape, particularly because APC, a hallmark early driver gene in colorectal cancer, was not included in this panel. Therefore, our assessment of clonality and shared/unique mutations should be interpreted as being limited to pathogenic mutations detected within the 22-gene panel, rather than as a comprehensive analysis of the entire truncal architecture of colorectal cancer. Nevertheless, this panel included several clinically relevant colorectal cancer-related driver genes, such as TP53, KRAS, PIK3CA, SMAD4, and BRAF, and allowed paired comparison of driver-level alterations between treatment-naïve primary and locally recurrent lesions.

In conclusion, our findings demonstrate that LRRC is not merely residual disease but an evolutionarily advanced tumor characterized by both the persistence and dominance of truncal drivers and the acquisition of clinically relevant alterations. In particular, the presence of potentially actionable alterations involving MET and FGFR family genes in recurrent lesions underscores that molecular profiling of recurrent tumors may provide opportunities to guide therapeutic strategies in this challenging disease.

## Supplementary Information

Below is the link to the electronic supplementary material.

Supplementary file1 (XLS 222 KB)

Supplementary file2 (XLSX 14 KB)

Supplementary file3 (XLS 137 KB)

Supplementary file4 (XLSX 25 KB)

Supplementary file5 (XLSX 32 KB)

Supplementary file6 (XLS 68 KB)

Supplementary file7 (XLS 86 KB)

Supplementary file8 (XLS 55 KB)

Supplementary file9 (XLS 87 KB)

Supplementary file10 (XLS 81 KB)

Supplementary file11 (XLS 101 KB)

Supplementary file12 (XLS 37 KB)

Supplementary file13 (XLS 52 KB)

Supplementary file14 (XLSX 54 KB)

Supplementary file15 (XLS 97 KB)

Supplementary file16 (TIF 103 KB)

Supplementary file17 (TIF 105 KB)

Supplementary file18 (XLSX 78 KB)

Supplementary file19 (XLSX 31 KB)

Supplementary file20 (XLS 432 KB)

Supplementary file21 (XLS 231 KB)

Supplementary file22 (XLS 69 KB)

Supplementary file23 (XLS 238 KB)

Supplementary file24 (XLS 222 KB)

Supplementary file25 (XLS 213 KB)

Supplementary file26 (XLS 81 KB)

Supplementary file27 (XLSX 14 KB)

Supplementary file28 (XLSX 15 KB)

Supplementary file29 (XLS 65 KB)

Supplementary file30 (XLS 352 KB)

Supplementary file31 (XLS 158 KB)

Supplementary file32 (XLS 98 KB)

## References

[1] Sung H, Siegel RL, Laversanne M, Jiang C, Morgan E, Zahwe M, et al. Colorectal cancer incidence trends in younger versus older adults: an analysis of population-based cancer registry data. Lancet Oncol. 2025;26:51–63. 10.1016/S1470-2045(24)00600-4. PMC11695264 39674189

[2] Sauer R, Becker H, Hohenberger W, Rodel C, Wittekind C, Fietkau R, et al. Preoperative versus postoperative chemoradiotherapy for rectal cancer. N Engl J Med. 2004;351(17):1731–40. 10.1056/NEJMoa040694. 15496622

[3] Jayne DG, Thorpe HC, Copeland J, Quirke P, Brown JM, Guillou PJ. Five-year follow-up of the medical research council CLASICC trial of laparoscopically assisted versus open surgery for colorectal cancer. Br J Surg. 2010;97:1638–45. 10.1002/bjs.7160. 20629110

[4] van Gijn W, Marijnen CA, Nagtegaal ID, Kranenbarg EM, Putter H, Wiggers T, et al. Preoperative radiotherapy combined with total mesorectal excision for resectable rectal cancer: 12 year follow-up of the multicentre, randomised controlled TME trial. Lancet Oncol. 2011;12:575–82. 10.1016/S1470-2045(11)70097-3. 21596621

[5] Dijkstra EA, Nilsson PJ, Hospers GAP, Bahadoer RR, Meershoek-Klein Kranenbarg E, Roodvoets AGH, et al. Locoregional failure during and after short-course radiotherapy followed by chemotherapy and surgery compared with long-course chemoradiotherapy and surgery: a 5-year follow-up of the RAPIDO trial. Ann Surg. 2023;278:e766–72. 10.1097/SLA.0000000000005799. PMC10481913 36661037

[6] Westberg K, Palmer G, Hjern F, Holm T, Martling A. Population-based study of surgical treatment with and without tumour resection in patients with locally recurrent rectal cancer. Br J Surg. 2019;106:790–8. 10.1002/bjs.11098. 30776087

[7] Hagemans JAW, Rees JMv, Alberda WJ, Rothbarth J, Nuyttens JJME, Meerten E, et al. Locally recurrent rectal cancer; long-term outcome of curative surgical and non-surgical treatment of 447 consecutive patients in a tertiary referral centre. Eur J Surg Oncol. 2020;46:448–54. 10.1016/j.ejso.2019.10.037. 31761506

[8] Heald RJ, Husband EM, Ryall RD. The mesorectum in rectal cancer surgery–the clue to pelvic recurrence? Br J Surg. 1982;69:613–6. 10.1002/bjs.1800691019. 6751457

[9] Maurer CA, Renzulli P, Kull C, Kaser SA, Mazzucchelli L, Ulrich A, et al. The impact of the introduction of total mesorectal excision on local recurrence rate and survival in rectal cancer: long-term results. Ann Surg Oncol. 2011;18:1899–906. 10.1245/s10434-011-1571-0. 21298350

[10] Uemura M, Ikeda M, Yamamoto H, Kitani K, Tokuoka M, Matsuda K, et al. Clinicopathological assessment of locally recurrent rectal cancer and relation to local re-recurrence. Ann Surg Oncol. 2011;18:1015–22. 10.1245/s10434-010-1435-z. 21082358

[11] Nowell PC. The clonal evolution of tumor cell populations. Science. 1976;194:23–8. 10.1126/science.959840. 959840

[12] Gerstung M, Jolly C, Leshchiner I, Dentro SC, Gonzalez S, Rosebrock D, et al. The evolutionary history of 2658 cancers. Nature. 2020;578:122–8. 10.1038/s41586-019-1907-7. PMC7054212 32025013

[13] Akagi T, Inomata M, Fujishima H, Fukuda M, Konishi T, Tsukamoto S, et al. Preoperative chemoradiotherapy versus surgery alone for advanced low rectal cancer: a large multicenter cohort study in Japan. Surg Today. 2020;50:1507–14. 10.1007/s00595-020-02034-2. 32524272

[14] Uemura M, Ikeda M, Sekimoto M, Haraguchi N, Mizushima T, Yamamoto H, et al. Prevention of severe pelvic abscess formation following extended radical surgery for locally recurrent rectal cancer. Ann Surg Oncol. 2009. 10.1245/s10434-009-0505-6. 19506961

[15] Ogawa H, Uemura M, Nishimura J, Hata T, Ikenaga M, Takemasa I, et al. Preoperative chemoradiation followed by extensive pelvic surgery improved the outcome of posterior invasive locally recurrent rectal cancer without deteriorating surgical morbidities: a retrospective, single-institution analysis. Ann Surg Oncol. 2015;22:4325–34. 10.1245/s10434-015-4452-0. 25893408

[16] Chatzopoulos K, Kotoula V, Koliou GA, Giannoulatou E, Papadopoulou K, Karavasilis V, et al. Genotype-phenotype associations in colorectal adenocarcinomas and their matched metastases. Hum Pathol. 2021;107:104–16. 10.1016/j.humpath.2020.10.009. 33161028

[17] Wang K, Li M, Hakonarson H. ANNOVAR: functional annotation of genetic variants from high-throughput sequencing data. Nucleic Acids Res. 2010;38:e164. 10.1093/nar/gkq603. PMC2938201 20601685

[18] Landrum MJ, Lee JM, Benson M, Brown GR, Chao C, Chitipiralla S, et al. ClinVar: improving access to variant interpretations and supporting evidence. Nucleic Acids Res. 2018;46:D1062–7. 10.1093/nar/gkx1153. PMC5753237 29165669

[19] Forbes S, Clements J, Dawson E, Bamford S, Webb T, Dogan A, et al. Cosmic 2005. Br J Cancer. 2006;94:318–22. 10.1038/sj.bjc.6602928. PMC2361125 16421597

[20] Adzhubei IA, Schmidt S, Peshkin L, Ramensky VE, Gerasimova A, Bork P, et al. A method and server for predicting damaging missense mutations. Nat Methods. 2010;7:248–9. 10.1038/nmeth0410-248. PMC2855889 20354512

[21] Roth A, Khattra J, Yap D, Wan A, Laks E, Biele J, et al. PyClone: statistical inference of clonal population structure in cancer. Nat Methods. 2014;11:396–8. 10.1038/nmeth.2883. PMC4864026 24633410

[22] Coombs CC, Gillis NK, Tan X, Berg JS, Ball M, Balasis ME, et al. Identification of clonal hematopoiesis mutations in solid tumor patients undergoing unpaired next-generation sequencing assays. Clin Cancer Res. 2018;24:5918–24. 10.1158/1078-0432.CCR-18-1201. PMC6812550 29866652

[23] Bobin C, Iddir Y, Butterworth C, Masliah-Planchon J, Saint-Charles A, Bellini A, et al. Sequential analysis of cfDNA reveals clonal evolution in patients with neuroblastoma receiving ALK-targeted therapy. Clin Cancer Res. 2024;30:3316–28. 10.1158/1078-0432.CCR-24-0753. PMC11292203 38787533

[24] PelvEx, C. Factors affecting outcomes following pelvic exenteration for locally recurrent rectal cancer. Br J Surg. 2018;105:650–7. 10.1002/bjs.10734. 29529336

[25] Detering R, Karthaus EG, Borstlap WAA, Marijnen CAM, van de Velde CJH, Bemelman WA, et al. Treatment and survival of locally recurrent rectal cancer: a cross-sectional population study 15 years after the Dutch TME trial. Eur J Surg Oncol. 2019;45:2059–69. 10.1016/j.ejso.2019.06.016. 31230980

[26] Braeckman N, Kerckhove E, Denys A, Sys G, van Ramshorst GH. Oncological and functional outcomes after sacrectomy for colorectal cancer: a systematic review. Colorectal Dis. 2025;27:e70083. 10.1111/codi.70083. 40185644

[27] Kapiteijn E, Marijnen CA, Nagtegaal ID, Putter H, Steup WH, Wiggers T, et al. Preoperative radiotherapy combined with total mesorectal excision for resectable rectal cancer. N Engl J Med. 2001;345:638–46. 10.1056/NEJMoa010580. 11547717

[28] Garcia-Aguilar J, Patil S, Gollub MJ, Kim JK, Yuval JB, Thompson HM, et al. Organ preservation in patients with rectal adenocarcinoma treated with total neoadjuvant therapy. J Clin Oncol. 2022;40:2546–56. 10.1200/jco.22.00032. PMC9362876 35483010

[29] Bahadoer RR, Dijkstra EA, Etten B, Marijnen CAM, Putter H, Kranenbarg EM-K, et al. Short-course radiotherapy followed by chemotherapy before total mesorectal excision (TME) versus preoperative chemoradiotherapy, TME, and optional adjuvant chemotherapy in locally advanced rectal cancer (RAPIDO): a randomised, open-label, phase 3 trial. Lancet Oncol. 2021;22:29–42. 10.1016/s1470-2045(20)30555-6. 33301740

[30] Yates LR, Campbell PJ. Evolution of the cancer genome. Nat Rev Genet. 2012;13:795–806. 10.1038/nrg3317. PMC3666082 23044827

[31] Gerlinger M, Rowan AJ, Horswell S, Math M, Larkin J, Endesfelder D, et al. Intratumor heterogeneity and branched evolution revealed by multiregion sequencing. N Engl J Med. 2012;366:883–92. 10.1056/nejmoa1113205. PMC4878653 22397650

[32] Yaeger R, Chatila WK, Lipsyc MD, Hechtman JF, Cercek A, Sanchez-Vega F, et al. Clinical sequencing defines the genomic landscape of metastatic colorectal cancer. Cancer Cell. 2018;33:125-136 e123. 10.1016/j.ccell.2017.12.004. PMC5765991 29316426

[33] Bardelli A, Corso S, Bertotti A, Hobor S, Valtorta E, Siravegna G, et al. Amplification of the MET receptor drives resistance to anti-EGFR therapies in colorectal cancer. Cancer Discov. 2013;3:658–73. 10.1158/2159-8290.CD-12-0558. PMC4078408 23729478

[34] Helsten T, Elkin S, Arthur E, Tomson BN, Carter J, Kurzrock R. The FGFR landscape in cancer: analysis of 4853 tumors by next-generation sequencing. Clin Cancer Res. 2016;22:259–67. 10.1158/1078-0432.CCR-14-3212. 26373574

[35] Vakiani E, Janakiraman M, Shen R, Sinha R, Zeng Z, Shia J, et al. Comparative genomic analysis of primary versus metastatic colorectal carcinomas. J Clin Oncol. 2012;30:2956–62. 10.1200/JCO.2011.38.2994. PMC3417049 22665543

